# From local validation to global standardization of three-dimensional printing in radiotherapy^☆^

**DOI:** 10.1016/j.phro.2026.101053

**Published:** 2026-07-31

**Authors:** José Vedelago, Christina Stengl, Barbara Knäusl

**Affiliations:** a Department of Medical Physics in Radiation Oncology, German Cancer Research Center (DKFZ), Im Neuenheimer Feld 280, Heidelberg, 69120, Germany; b Heidelberg Institute for Radiation Oncology (HIRO), National Center for Radiation Research in Oncology (NCRO), Heidelberg, 69120, Germany; c Department of Radiation Oncology, Heidelberg University Hospital (UKHD), Im Neuenheimer Feld 400, Heidelberg, 69120, Germany; d Department of Radiation Oncology, Medical University of Vienna, Währinger Gürtel 18-20, Vienna, 1090, Austria

Three-dimensional (3D) printing is exponentially gaining relevance across research fields. Particularly in radiation oncology, 3D printing offers a wide range of solutions from research and dosimetry to clinical applications. Such solutions include the design and fabrication of patient-specific immobilization devices, patient-specific boluses to enhance surface dose for skin cancer treatment, simple phantoms for quality assurance or complex anthropomorphic phantoms for dedicated studies, custom range modulators for proton therapy, dosimetric equipment and support devices for research experiments [1], [2], [3], [4], [5], [6], [7], [8], [9]. As the field continues to evolve, the question is no longer whether 3D printing can be used in radiotherapy, but whether its products can be consistently trusted across institutions and over long time periods. Unlike commercially manufactured radiotherapy devices, 3D-printed objects are often produced locally, using different 3D printer models, 3D-printed materials, slicer software settings, post-processing workflows, and characterized using different imaging protocols. Different centers may print nominally identical objects but obtain different geometric, mechanical, or radiological properties. What might seem insignificant variations in infill patterns, layer height, print orientation, or filament batch can alter the density of the 3D-printed object, geometric accuracy, computed tomography (CT) imaging contrast, and ultimately, its performance during dosimetric measurements. Moreover, these variations affect outcomes differently depending on the radiation type; for example, other aspects need to be considered for proton and ion beam therapy compared to photon therapy.

Current quality assurance (QA) approaches for 3D-printed objects are highly heterogeneous and not at all standardized for applications in radiation therapy. Some groups reported geometric accuracy through caliper measurements or CT-based comparisons, while others focused on agreement of dosimetric measurements, material characterization, or reproducibility assessments [10], [11], [12], [13], [14], [15], [16]. Acceptance criteria vary considerably, and many studies develop center-specific validation procedures that are difficult to compare with published work elsewhere. The field would greatly benefit from consensus-based QA recommendations and guidelines on how to implement them for radiotherapy [17], [18].

Jones et al. [19] published an audit of 3D-printed objects for use in radiotherapy, with a particular focus on testing the geometric accuracy of the printed samples and the water equivalence of the materials for photon beams. They reported an inter-center comparison involving ten institutions using three cuboid blocks specifically designed for the audit. CT numbers and mass densities showed relevant variations between the centers, with differences up to 200 Hounsfield units and 0.2 g/cm${}^{3}$, dosimetry validation measurements agreed within 2 standard deviations of the respective means. The stereolithography (STL) files of the designed objects [20] that were published as part of thework enable any other institution to cross-check the geometric accuracy of their 3D printing and water equivalence by following the steps presented in the work by Jones et al. [19].

Beyond the production aspects investigated by Jones et al. [19], the long-term stability of the materials should also be considered, particularly for routine QA measurements and research applications. Material properties may evolve over time as a result of storage conditions and cumulative radiation exposure, potentially affecting their dosimetric performance. For several aspects, photon and particle irradiation might require varying properties and different QA measures [17], [18].

Perhaps the most significant opportunity to advance the use of 3D printed equipment in radiotherapy lies in data sharing (Fig. 1). Every institution implementing 3D printing generates valuable QA data that often remains confined to local reports, supplementary material, or internal documentation. Collectively, these measurements represent a potentially powerful resource for the community. A multi-institutional repository containing printer specifications, materials, print settings, geometric measurements, CT characteristics, density data, and dosimetry validation results could accelerate standardization efforts substantially. For this, reporting recommendations and guidelines are available [17], [18]. Such a database would enable benchmarking across centers, identification of systematic biases, and development of evidence-based tolerance levels. Over time, it could support the creation of reference datasets for commonly used materials and devices, reducing duplication of effort while improving confidence in clinical implementation.

As 3D printing becomes increasingly adopted in radiotherapy, thorough QA procedures will contribute towards reproducible and comparable research, facilitating future developments and ultimately improving the treatments for our patients.

## Declaration of generative AI

**Fig. 1:**
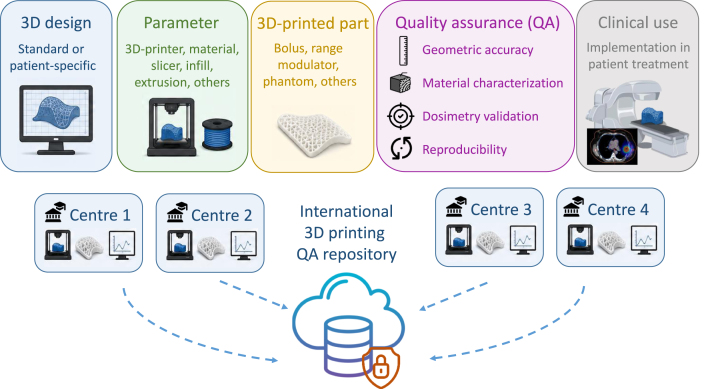
Illustration on how data sharing could support an international 3D printing repository for QA of the 3D-printed objects for their use in radiation therapy.

For the generation of the icons in Fig. 1, the authors used ChatGPT. After using this tool, the authors reviewed the icons and take full responsibility for the content of the published article.

## Declaration of competing interest

The authors declare that they have no known competing financial interests or personal relationships that could have appeared to influence the work reported in this paper.

José Vedelago is an Associate Editor for Physics and Imaging in Radiation Oncology. Barbara Knäusl is an Editor-in-Chief for Physics and Imaging in Radiation Oncology. The article will be published as correspondence and, as such, will not undergo a formal review process.
